# A novel *in vitro* 3D model of the human bone marrow to bridge the gap between *in vitro* and *in vivo* genotoxicity testing

**DOI:** 10.1093/mutage/geac009

**Published:** 2022-04-08

**Authors:** Alexander R Vernon, Roy M Pemberton, H Ruth Morse

**Affiliations:** Department of Applied Sciences, University of the West of England, Bristol, Frenchay Campus, Coldharbour Lane, Bristol BS16 1QY, United Kingdom

**Keywords:** genotoxicity testing, micronucleus assay, novel model, *in vitro*, bone marrow

## Abstract

The regulatory 2D *in vitro* micronucleus (MN) assay is part of a battery of tests, used to test for genotoxicity of new and existing compounds before they are assessed *in vivo* (ICH S2). The 2D MN assay consists of a monolayer of cells, whereas the *in vivo* bone marrow (BM) setting comprises a multicellular environment within a three-dimensional extracellular matrix. Although the *in vitro* MN assay follows a robust protocol set out by the Organisation for Economic Co-operation and Development (OECD) to comply with regulatory bodies, some compounds have been identified as negative genotoxicants within the *in vitro* MN assay but marginally positive when assessed *in vivo*. The glucocorticoids, which are weakly positive *in vivo*, have generally been suggested to pose no long-term carcinogenic risk; however, for novel compounds of unknown activity, improved prediction of genotoxicity is imperative. To help address this observation, we describe a novel 3D *in vitro* assay which aims to replicate the results seen within the *in vivo* BM microenvironment. AlgiMatrix scaffolds were optimized for seeding with HS-5 human BM stromal cells as a BM microenvironment, to which the human lymphoblast cell line TK6 was added. An MN assay was performed aligning with the 2D regulatory assay protocol. Utilizing this novel 3D *in vitro* model of the BM, known genotoxicants (mitomycin C, etoposide, and paclitaxel), a negative control (caffeine), and *in vivo* positive glucocorticoids (dexamethasone and prednisolone) were investigated for the induction of MN. It was found, in agreement with historical *in vivo* data, that the model could accurately predict the *in vivo* outcome of the glucocorticoids, unlike the regulatory 2D *in vitro* MN assay. These preliminary results suggest our 3D MN assay may better predict the outcome of *in vivo* MN tests, compared with the standard 2D assay.

## Introduction

The human bone marrow (BM) is one of the largest organs, accounting for around 5% of the total body weight, consisting primarily of haematologically active red marrow [1]. This active marrow induces constant proliferation and differentiation of haematopoietic stem cells (HSCs) into several blood cell lineages, through contact and support from mesenchymal stromal, osteoblast, and endothelial cells of the BM microenvironment residing upon a three-dimensional (3D) extracellular matrix (ECM) [2]. It is these 3D interactions that allow the successful homing, self-renewal, and differentiation of HSC and progeny cells [3]. Due to the delicate nature of this environment and proliferative pressure, the cells can be susceptible to genomic damage (genotoxicity) induced by prophylactic or therapeutic compounds [4]; the BM is thus a target organ for *in vivo* genotoxicity testing.

Within the pharmaceutical industry, in an effort to predict the effects that exposure to a newly developed compound will have on the BM cell population, the regulatory *in vitro*, followed by the *in vivo*, micronucleus (MN) assay is routinely used as a part of a battery of genotoxicity tests (ICH S2). These assays comply with regulatory bodies, using guidelines set out by the Organisation for Economic Co-operation and Development (OECD) [5]. The MN assay identifies the production of a second ‘micro’ nucleus away from the main nucleus through the loss or breakage of a chromosome during active mitosis [6]. The *in vitro* MN assay is first used to predict a compound’s genotoxicity, utilizing a single cell line culture of human or mouse lymphoblastic cells; the compound is then tested *in vivo*, where visualization of MN is assessed in developing erythrocytes of the rodent BM [7]. The 2D regulatory *in vitro* assay is meant to be predictive of the 3D  multicellular *in vivo* microenvironment; however, some inconsistency has been observed. For example, glucocorticoids such as dexamethasone and prednisolone have been used in the clinic safely with no known carcinogenicity, but were demonstrated to be marginally positive *in vivo* despite being negative for genotoxicity in the 2D regulatory *in vitro* MN assay [8]. This discrepancy in MN formation between the *in vitro* and *in vivo* conditions has been postulated to be due to changes in biological processes occurring *in vivo* (e.g. hypothermia, hyperthermia, increased erythropoiesis) not seen within the *in vitro* assay [9–11], however, without a comprehensive concept of the mechanisms involved, we must be cautious about *in vivo* positive outcomes.

As mentioned previously, the BM is a complex multicellular 3D environment, but this is not reproduced within the 2D monoculture used routinely *in vitro*. Therefore, the development of a simple 3D *in vitro* multicellular model could aid understanding of the mechanisms behind these *in vivo* positives, more accurately predict the *in vivo* genotoxicity of new and existing pharmaceuticals, and help determine what is, and is not, safe.

In this study, we describe the development of a simple and reproducible co-culture model of the human BM upon a 3D artificial ECM, and utilizing the same timeframe as the 2D regulatory *in vitro* MN assay, we examined the genotoxicity and cytotoxicity of three known genotoxicants (mitomycin C [MMC], etoposide, and paclitaxel), two compounds weakly positive *in vivo* (dexamethasone and prednisolone) and one toxic but non-carcinogenic compound (caffeine) [12]. The data obtained from each compound within the *in vitro* 3D model, were compared with results obtained in the 2D *in vitro* MN assay and historical rodent *in vivo* data, for the 3D model’s ability to accurately predict *in vitro* the level of MN produced *in vivo*.

## Materials and methods

### Chemicals

Reagents and test chemicals were purchased from Sigma-Aldrich unless stated otherwise. MMC, etoposide, paclitaxel, dexamethasone, and prednisolone were dissolved and diluted in dimethyl sulphoxide (DMSO; final concentration 0.01%) (Thermo Fisher Scientific), whereas caffeine was dissolved and diluted in dH_2_O.

### Cell culture

The human lymphoblastoid cell line, TK6 (13051501; ECACC) and human BM stromal cell line, HS-5 (CRL-11882; ATCC), was cultured in RPMI 1640 medium (Life Technologies) supplemented with 10% heat-inactivated foetal bovine serum (Life Technologies), 2 mM L-glutamine, 100 U/ml penicillin, and 100 mg/ml streptomycin (‘complete medium’). TK6 cells were maintained in culture between 3 × 10^5^ and 1 × 10^6^ cells/ml, and HS-5 cells were maintained between 4 × 10^3^ and 1.3 × 10^4^ cells/cm^2^ (37°C, 5% CO_2_). HS-5 and TK6 cells were cultured for 1 week prior to experimentation.

### Cell counts

Cell counts within both the 2D and 3D assays were assessed using a 1:1 dilution of 0.4% trypan blue (TB) to cells. The sample was then analysed using a Luna-FL™ Automated Cell Counter (Labtech International Ltd).

### Flow cytometry

Cells were fluorescently labelled for expression of the proliferation marker Ki67 (1:20 dilution, mouse anti-human IgG1 PE-conjugated, 5 µl per test; 12-5699-42, Thermo Fisher Scientific), and cell surface markers CD19 (1:20 dilution, mouse anti-human IgG1 FITC-conjugated, 20 µl per test; 555412 BD Biosciences) and CD20 (1:20 dilution, mouse anti-human IgG2b PerCP-cy 5.5 conjugated, 5 µl per test; 560736 BD Biosciences). Cells were assessed using flow cytometry with a gating strategy to isolate single cells and exclude doublets and cellular debris. The population of interest was first identified using a side scatter (SSC) area vs. forward scatter (FSC) area plot discarding debris or cellular remnants. Further gating by granulation (SSC area vs. SSC height) and then size (FSC area vs. FSC height) reduced contamination from doublets before assessing the population for the presence or absence of antibody within 50 000 events after initial gating. Flow cytometry was conducted on a Fortessa utilizing the FACSDiva software (Becton Dickinson [BD], New Jersey).

### Characterization of AlgiMatrix™ 3D bioscaffold internal structure

The AlgiMatrix™ 3D culture system (Thermo Fisher Scientific), an alginate-derived scaffold, was obtained as a freeze-dried sponge. Dry AlgiMatrix™ scaffolds were solidified (or ‘firmed’) by suspension in 500 µl of either a 50%, 25%, or 10% solution of AlgiMatrix™ firming buffer: complete medium (without HS-5 cells) for ~5 min in sterile culture wells. Rehydrated scaffolds without HS-5 cells were  visualized via scanning electron microscopy (SEM) for pore size at differing firming buffer concentrations. Briefly, each scaffold was placed on lint-free tissue overnight to draw out any moisture within the scaffold. The dehydrated scaffold was then placed on a carbon conductive tab on pin stubs (TAAB, UK), and sputter coated with gold utilizing a Emscope SC 500 sputter coater (Bio-Rad, Watford, UK). Imagining of pore size was then achieved using a FEI Quanta 650 field emission SEM.

### Optimization of HS-5 seeding onto AlgiMatrix™ scaffolds

Rehydrated scaffolds were washed with 1 ml of the complete medium before seeding cells and subsequent analysis by light microscope for cellular distribution. HS-5 cells were seeded into 50% firmed AlgiMatrix™ scaffolds at a density of 2.5, 5, 7.5, and 10 × 10^5^ cells/scaffold and placed in a 12-well plate with 3 ml of fresh complete medium. Scaffolds were incubated for an initial 24 h, a 100% medium change conducted, and then incubated for a further 312 h with a 50% medium change every 48 h. Representative scaffolds were harvested at 48-h time points over this 312-h period. At each time point, cell number (utilizing Luna-FL™ Counter), the presence of Ki67, and stages of the cell cycle (propidium iodide [PI]) were assessed.

Once an appropriate seeding density of HS-5 onto the AlgiMatrix™ scaffold had been identified, long-term culture conditions of HS-5-seeded AlgiMatrix™ scaffolds were investigated. HS-5 cells (2.5 × 10^5^ cells total), in 500 µl of either 50%, 25%, or 10% firming buffer in complete medium were seeded onto dry AlgiMatrix™ scaffolds and left for ~5 min. The scaffolds were then either washed with complete medium or left unwashed, before being transferred into 12-well plates; 3 ml of complete medium were then added and scaffolds cultured for an initial 24 h. Scaffolds then underwent either a 100%, 50%, or 0% complete medium change every subsequent 48 h during culture. At 24-h intervals, samples of HS-5-cultured scaffolds were transferred into 15 ml centrifuge tubes, 2 ml of AlgiMatrix™ dissolving buffer was added and left for <30 min with gentle agitation every 2 min until the scaffold had dissolved. Cells were collected by centrifugation (300×*g*, 7 min), the supernatant removed, fresh medium added, and an aliquot was taken for cell number and viability assessment.

### Optimization of the addition of TK6 cells onto HS-5-seeded AlgiMatrix™ scaffolds

HS-5 cells were cultured within AlgiMatrix™ scaffolds for 144 h in 12-well plates at the optimized concentration and medium regimen previously identified. TK6 cells were directly added to the scaffold at 0.5, 1, or 3.5 × 10^5^ cells/ml in complete medium, mixed by pipetting, and left to incubate for 102 h. A 50% or 0% medium change was conducted every 0, 24, or 48 h over this 102-h period. The cells were collected twice a day from dissolved scaffolds and the medium for counting; sampling was 6 hours apart. Cells were washed in phosphate-buffered saline (PBS) before being fluorescently labelled and assessed for CD19 positivity via flow cytometry to identify TK6 cells.

To determine if HS-5 viability was influenced by the addition of TK6 cells after the initial incubation, TK6 cells were seeded into a 0.25 µm pore well insert (Millipore), at a concentration of 0.5 × 10^5^ cells/ml, and inserts were placed into wells (12-well plates) containing HS-5-seeded AlgiMatrix™ scaffolds. The well insert allowed cellular communication but prevented physical contact of HS-5 cells with TK6 cells. A 50% medium replacement was then conducted at either 24- or 48-h intervals over a 96-h period. Well inserts were removed, and AlgiMatrix™ scaffolds dissolved every 48 h; HS-5 cells were counted, stained with the fixable viability dye eFluor 520 (Thermo Fisher Scientific), and assessed via flow cytometry. Negative control dead (HS-5 incubated at 100°C for 10 min), positive control live (HS-5 cells at a viability of >90%), and 50:50 (positive:negative) control for percentage positivity were also assessed. The percentage positive and total cell number were then used to calculate the total number of live HS-5 cells.

### Final AlgiMatrix™ 3D culture model

Prior to culturing within the AlgiMatrix™ 3D culture system, HS-5 cells were resuspended at a final density of 5 × 10^5^ cells/ml in a 1:1 dilution of culture medium: firming buffer (final conc. 50% v/v). A 500 µl aliquot of cell suspension was then added to each AlgiMatrix™ scaffold (2.5 × 10^5^ cells per scaffold) and incubated for 5 min. The scaffold was washed with complete medium, transferred to a 12-well plate, and 3 ml of complete medium was added before a 24-h incubation (37°C, 5% CO_2_). After 24 h, a complete medium change was conducted, and scaffolds were incubated for a further 144 h with a demi-depletion of medium (with fresh medium replacement) every 48 h. Following this, the medium was removed from around the AlgiMatrix™ and replaced with 3 ml of TK6 cells at an initial density of 0.5 × 10^5^ cells/ml. A demi-depletion with medium replacement was conducted every 24 h over a 72-h period, at which point TK6 cells were in their exponential phase for the addition of the test compound.

### Selection of doses for 2D and 3D study

Final concentrations of test compounds for the 2D *in vitro* MN assay were selected based on the criteria set out in OECD guideline 487 [13], which requires doses leading to a relative population doubling (RPD) of >55% ± 5%. As an RPD could not be determined within the 3D model, doses were increased for each compound within the 2D *in vitro* MN assay to identify RPDs of <50%. Doses of the compound that achieved an RPD within the 2D assay of 100%–80%, 80%–60%, 60%–40%, 40%–20%, 20%–0% were used within the 3D model. Compounds were tested to either maximum soluble concentration or to the highest dose of 1 mM (where solubility exceeded 1 mM). Compounds that produced a >50% RPD within the 2D assay up to the maximum doses were then assessed at all doses for that compound during the 3D assay.

### Genotoxicity assessment: the micronucleus assays following standard 2D culture and the 3D AlgiMatrix™ scaffold model

**Figure 1.**
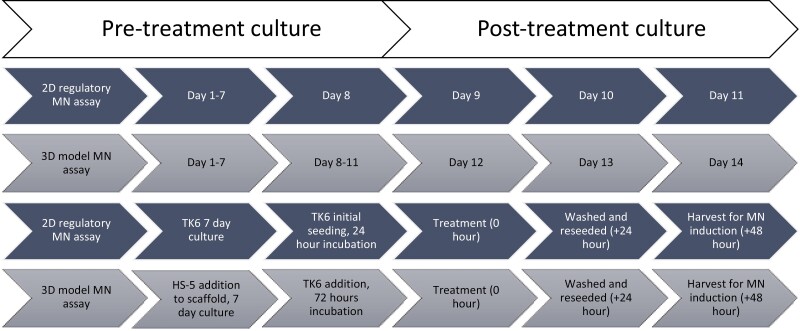
A comparison of timelines between the MN assay conducted within the 3D model and 2D regulatory assay.

The extent of chromosome alterations induced by each compound dose was analysed using the *in vitro* MN assay. The MN assay protocol in both 2D and 3D cultures can be seen in Fig. 1; both approaches were over a maximum 14-day period to ensure that TK6 cells were in their exponential phase for genotoxicity assessment. As described in the ‘Cell culture’ section, an initial pre-treatment culture period of either 8 (2D MN assay) or 11 days (3D MN assay) was performed, followed by a 24-h treatment + 24-h recovery period approach [14]. After 24-h treatment, cells within the medium of both the 2D assay and 3D model were washed and reseeded into a new flask or original well, respectively. After 24-h recovery, MN were identified in both 2D and 3D by centrifuging cells onto glass slides utilizing a cytospin 3 (Thermo Fisher Scientific) at 800 rpm for 8 min; slides were fixed in 100% methanol (10 min, room temperature), stained for 1 min with acridine orange (AO) (12 mg/100 ml PBS, room temperature), then washed for 10 and 15 min in PBS, and allowed to dry. MN were identified using a Nikon Eclipse TE300 microscope with an attached Nikon Coolpix 950 camera at 40× magnification. MN were scored in a total of 2000 mononucleated cells per 2D treatment. Doses that induced an RPD <50% were not scored for MN. A total of 2000 mononucleated cells were analysed for MN in both the medium and the scaffold of the 3D culture (overall total 4000 cells). For both 2D and 3D assays, three biological replicates were performed; data were averaged and presented per 1000 mononucleated cells. RPD [15] was measured within the 2D MN assay, relative to the vehicle (0.01% DMSO or H_2_O) and positive (MMC) control in line with OECD guideline 487 [13]. Cell counts were also taken of pre-treatment ‘baseline’ cells within the medium and scaffold to confirm cellular proliferation.

### Statistical analysis

All values are presented as mean ± SD of three biological repeats, unless otherwise stated. All statistical analysis and graphical illustrations were conducted using GraphPad Prism 7.0 software. Firstly, all samples were analysed for normal distribution using the Shapiro–Wilk test. A two-way ANOVA was used to compare samples for simple effects within rows, followed by a Dunnett’s test to identify pairs with significant differences. A one-way ANOVA, followed by a Dunnett’s test, was also used to identify significant increases in MN from the vehicle control. A *P*-value of ≤.05 was considered significant.

## Results

### Developing a 3D model of the bone marrow

To develop a model which simulates the BM microenvironment, a commercial scaffold AlgiMatrix™, a hydrogel made of biologically inactive alginate, was used for its ability to incorporate and retrieve cells within the scaffold itself. However, optimization was required in order to simulate the complexities seen within the *in vivo* BM.

### Optimization of initial solidifying and eventual dissolving of AlgiMatrix™ scaffolds

**Figure 2.**
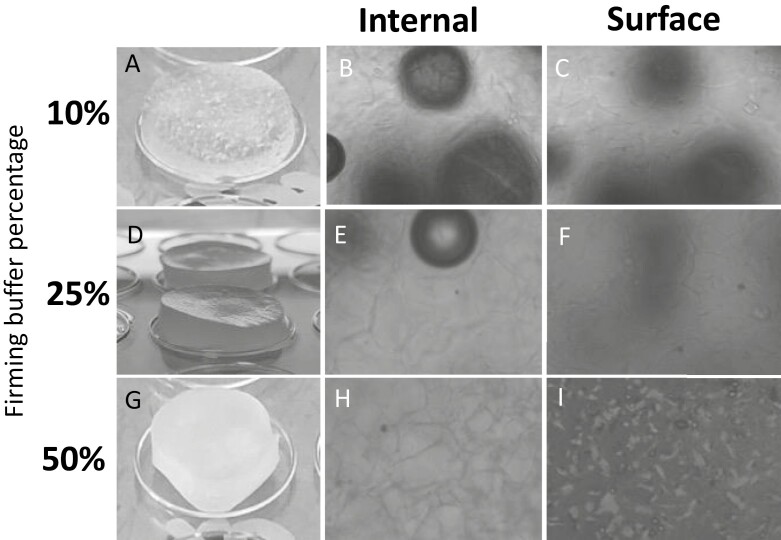
Images depicting the physical structural difference seen with increasing firming buffer concentration of AlgiMatrix™ scaffolds. A 10%, 25%, or 50% solution of firming buffer and medium was added to each dry AlgiMatrix™ scaffold for >5 min for solidification. The external appearance of each scaffold can be seen in A (10%), D (25%), and G (50%). Light microscopy (10× magnification) was then used to look at the internal (B, E, and H) and surface (C, F, and I) of each solid scaffold.

The commercial AlgiMatrix™ scaffold arrived as a freeze-dried alginate solution, which is rehydrated with differing concentrations of firming buffer dependent on the internal structure required. AlgiMatrix™ scaffolds were subjected to 10%, 25%, or 50% firming buffer in complete medium with the internal and external structure assessed by light microscopy (Fig. 2). The internal (Fig. 2B, E, and H) and external structure (Fig. 2C, F, and I) showed little to no microscopic appearance in either 10%, 25%, or 50% firming  conditions. However, the mechanical structure and consistency of a 10% scaffold (Fig. 2A) were much more gelatinous than that of 25% (Fig. 2D) or 50% (Fig. 2G) scaffold. The former fell apart when manipulated, whereas scaffolds solidified with 25% or 50% firming solution held their shape.

**Figure 3.**
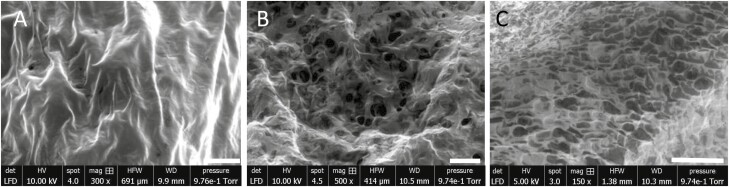
Scanning electron microscopy (SEM) images of AlgiMatrix™ scaffolds hardened with a 10%, 25%, or 50% firming buffer. A 10% (A), 25% (B), or 50% (C) solution of firming buffer and medium was added to each dry AlgiMatrix™ scaffold for >5 min, allowed to air dry for 24 h before being placed on lint-free tissue. Once residual medium had been drawn out, the scaffold was then pressurized within the SEM to evaporate further moisture before images were taken. (A) 300× magnification, (B) 500× magnification, and (C) 150× magnification. Scale bars are indicated by the white bar and represent 100 µm (A), 50 µm (B), and 300 µm (C).

SEM was used to identify finer microscopic differences at >150× magnification (Fig. 3). A 10% firming solution (Fig. 3A) produced a structure with no visible pores. However, scaffolds solidified with a 25% firming solution (Fig. 3B) gave pore sizes of around 25 µm and a 50% firming solution (Fig. 3C) gave a uniform pore size of ~120 µm.

### Effect of firming buffer on incorporated HS-5 cells and optimization of long-term culture conditions

**Figure 4.**
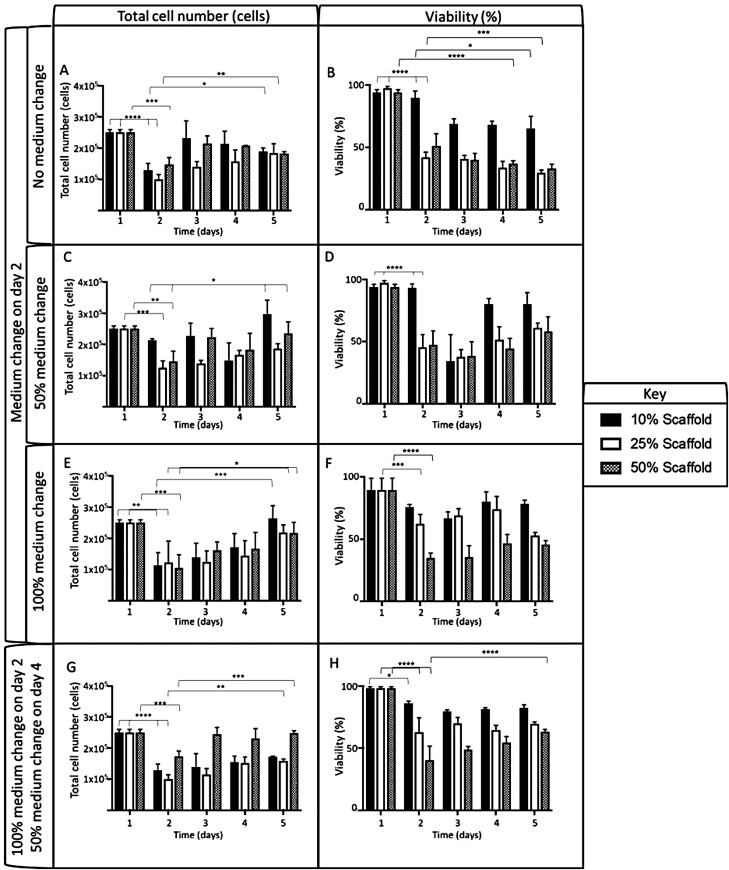
Cell number and viability of HS-5 cells seeded at a concentration of 2.5 × 10^5^ onto AlgiMatrix™ scaffolds. The cell number (A, E, C) and viability (B, D, F) of HS-5 cells seeded at a concentration of 2.5 × 10^5^ onto AlgiMatrix™ scaffolds, incubated for 5 days with a 0% (A, B), 50% (C, D), or 100% (E, F) medium change on Day 2. Cell number (G) and viability (H) were also taken for cells that underwent a 100% medium change on Day 2 and a 50% medium change on Day 4. In all experiments, HS-5 cells were seeded in a 10%, 25%, and 50% solidified AlgiMatrix™ scaffold for <5 min before the addition of fresh medium. At each time point, scaffolds were dissolved with viability and cell number assessed using an acridine orange and propidium iodide stain (*n* = 3). Significant differences between samples were calculated using a two-way ANOVA followed by a Dunnett’s test. The *P*-value is indicated by *P < .05, **P < .01, ***P < .001, ****P < .0001.

The effect of incorporating HS-5 cells into the scaffold itself with either a 10%, 25%, or 50% firming solution was investigated (Fig. 4). Once seeded, each scaffold contained 2.5 × 10^5^ total HS-5 cells and in 2 ml of fresh medium; these were incubated, and had either a 0%, 50%, or 100% medium change on Day 2, with cell number and viability determined using an AO/PI stain. Scaffolds treated with either 25% or 50% firming buffer demonstrated a statistically significant decrease in cell number (Fig. 4A, C, and E) and viability (Fig. 4B, D, and F) within the first 24 h. However, HS-5 cells incorporated into a 10% scaffold remained >90% viable (reductions in cell number can be seen before a 0% or 100% medium change in the same firming condition; Fig. 4A and E). Once a 0%, 50%, or 100% medium change had occurred on Day 2, the scaffold was incubated for a further 3 days. Those scaffolds which hadn’t had a medium change (Fig. 4A) saw a varying increase in cell number from Days 2 to 5 in scaffolds treated with a 10%, 25%, and 50% firming solution reaching similar numbers by Day 5. However, there was a statistical reduction in viability over the same time period in all conditions.

Scaffolds that underwent a 50% medium change on Day 2 (Fig. 4C and D), resulted in a statistical increase in cell number within the 10% and 50% firming solution groups. However, over the same time period (Days 2–5), there was no statistical increase in viability in all firming conditions. Finally, those cells which underwent a 100% medium change on Day 2 (Fig. 4E and F) had gradual and statistical increases in cell number (Fig. 4E) in all firming conditions over the 3-day period. In the same period, however, no significant increase in viability was seen in any of the firming conditions. This infers that the initial addition of firming buffer (evident at 25% and 50%; Fig. 4E) had a detrimental effect on HS-5 viability, however, the addition of a medium change on Day 2 reduced this effect.

To increase the longevity and viability of cells within the scaffold, the addition of a medium change on Day 4 was investigated (Fig. 4G and H). Scaffolds were treated in the same way as described previously but on Day 4, a further 50% change was performed. This protocol produced a statistical increase in cell number of those cells treated with a 25% or 50% firming solution between Days 2 and 5. However, only those cells within 50% treated scaffolds resulted in a statistical increase in viability over the same period.

These results identified that a 50% firming solution, 100% medium change on Day 2, and a 50% change on Day 4 gave a robust scaffold with maintenance of total cell number at >2.5 × 10^5^ and viability >50%. Therefore, this protocol was used to assess the optimal initial seeding density of HS-5 onto AlgiMatrix™ scaffold for long-term co-culture.

### Identifying the optimal seeding density of HS-5 cells within AlgiMatrix™ scaffolds

**Figure 5.**
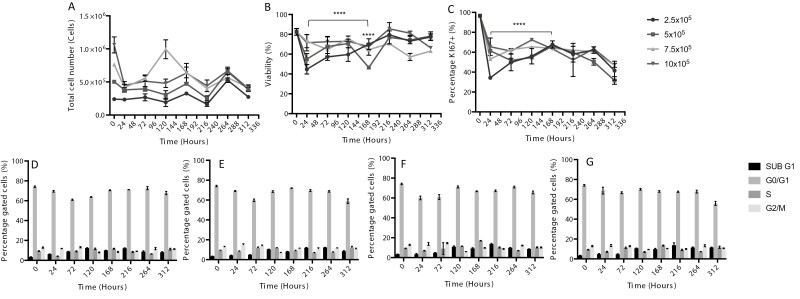
The growth of HS-5 cells seeded at 2.5, 5, 7.5, and 10 × 10^5^ cells onto an AlgiMatrix™ scaffold. HS-5 cells were seeded at a density of 2.5, 5, 7.5, and 10 × 10^5^ cells onto AlgiMatrix™ scaffolds, transferred to a 12-well plate, incubated for 24 h, complete medium change was undertaken and incubated for a further 312 h with a 50% medium change every 48 h. At each 48-h time point, cells were harvested and assessed for total cell number (A), viability (B), and the presence of Ki67 (C). Cells at a starting seeding density of 2.5 (D), 5 (E), 7.5 (F), and 10 × 10^5^ (G) cells were also analysed for cell cycle over the same period (50 000 events, *n* = 3). Significant differences were calculated using a two-way ANOVA followed by a Dunnett’s test. Significant differences between (B) 24 h, 2.5 vs. 168 h, 2.5**** and 2.5 vs. 5**** and (C) 24 h, 2.5 vs. 168 h, 2.5**** were ****P < .0001.

The growth of differing initial total cells seeded onto an AlgiMatrix™ and their effects on long-term culture was investigated. Scaffolds were seeded in a 12-well plate with either 2.5, 5, 7.5, or 10 × 10^5^ total HS-5 cells and assessed for cell number, viability, and the presence of Ki67 over a 312-h period (Fig. 5A–C). It can be seen that after seeding cells at 5, 7.5, and 10 × 10^5^ per scaffold, there were decreases in cell number, viability, and Ki67 over the first 24-h incubation period, with no significant difference between each. However, those scaffolds seeded with 2.5 × 10^5^ total cells did not show a reduction in cell number but did decrease in viability and Ki67 during the same time period. The following 144 h saw a statistical increase in viability (42%–70%) and Ki67 (38%–65%) only in those cells initially seeded at 2.5 × 10^5^ total cells, with total cell number over this period remaining constant at 2.5 × 10^5^ total cells. The rise in viability continued in the 2.5 × 10^5^ group but coincided with a decrease in Ki67. Those scaffolds initially seeded at 7.5 × 10^5^ total cells did show a spike in cell number (1 × 10^6^ cells) at 120 h, but this decreased over the remaining time along with both viability and Ki67. Cell cycle was also assessed for 2.5, 5, 7.5, and 10 × 10^5^ cells/scaffold (Fig. 5D–G), but no significant difference was seen between culture wells. This result reflected similar experiments conducted in 2D (results not shown) where a high level of cells with the G0/G1 transition was also maintained. These results indicate that a starting seeding density of 2.5 × 10^5^ HS-5 cells per scaffold left to incubate over a 168-h period would be suitable for the subsequent addition of TK6 cells.

### Optimal seeding density and identification of TK6 for optimal long-term co-culture conditions

**Figure 6.**
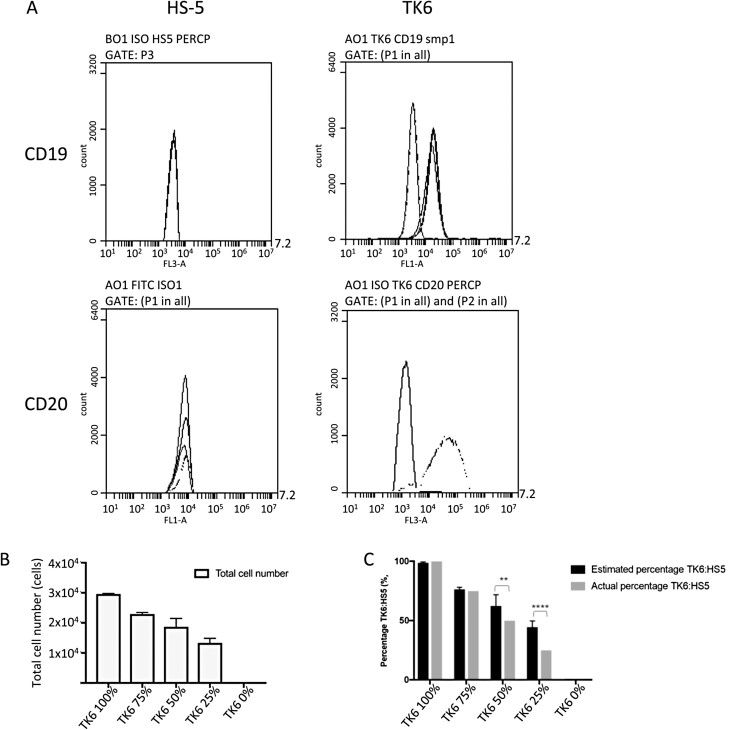
Histogram plot of CD19 and CD20 expression in TK6 and HS-5 cell lines. The expression of CD19 and CD20 (A) in TK6 and HS-5 cells. Bar chart showing the total number (B) and percentage (C) of TK6 cells expressing CD19 in differing ratios with HS-5 (50 000 events, *n* = 3). Significant differences between samples were calculated using a two-way ANOVA followed by a Dunnett’s test. The *P* values are indicated by **P < .01,  ****P < .0001.

In order to distinguish TK6 from HS-5 cells in future co-culture experimentation, the use of fluorescent labels for TK6-specific CD19 and CD20 markers was investigated. The CD19 and CD20 membrane markers were investigated for their presence on TK6 and HS-5 cells. HS-5 cells incubated with anti-CD19 and anti-CD20 antibodies only produced a peak with the isotype control when analysed by flow cytometry (Fig. 6A), demonstrating a lack of expression of either marker on these cells. As expected, TK6 cells produced a sharp peak for CD19 to the right of the isotype control consistent with high-level expression on all cells (Fig. 6A). However, a broader peak can be seen for CD20 as fewer TK6 cells express this marker and at more variable levels. Having validated CD19 as a highly expressed and reliable TK6 cell marker, the aim was to use CD19 expression to accurately identify and quantify TK6 cells in a mixture of TK6 and HS-5 cells, such that the approach could be used in future co-culture assays.

Mixtures of 3 × 10^5^ TK6 and HS-5 total cells in ratios of 100:0, 75:25, 50:50, 25:75, and 0:100 were stained with anti-CD19 antibodies and analysed by flow cytometry for the number of CD19-positive cells and percentage of TK6 vs. HS-5 in 50 000 cell events (Fig. 6B and C). Comparison of the percentage positive cells and the total cell number counted using TB (Fig. 6B) showed that at 100% or 3 × 10^5^ TK6 cells, 2.9 × 10^5^ cells were stained and detected; at 50% or 1.5 × 10^5^ TK6 cells, 1.9 × 10^5^ cells were stained and detected; and at 0% or 0 TK6 cells no cells were detected. These total cell numbers were then converted into a percentage. Figure 6C shows the comparison between the original ratio and the percentage of TK6 determined by flow cytometry in each sample. There was no significant difference between the two values for 100%, 75%, and 0% TK6 mixtures. However, the actual percentage (original TB-counted cell mixture) was statistically decreased compared to the estimated percentage (by flow cytometry) at 50% and 25% TK6.

**Figure 7.**
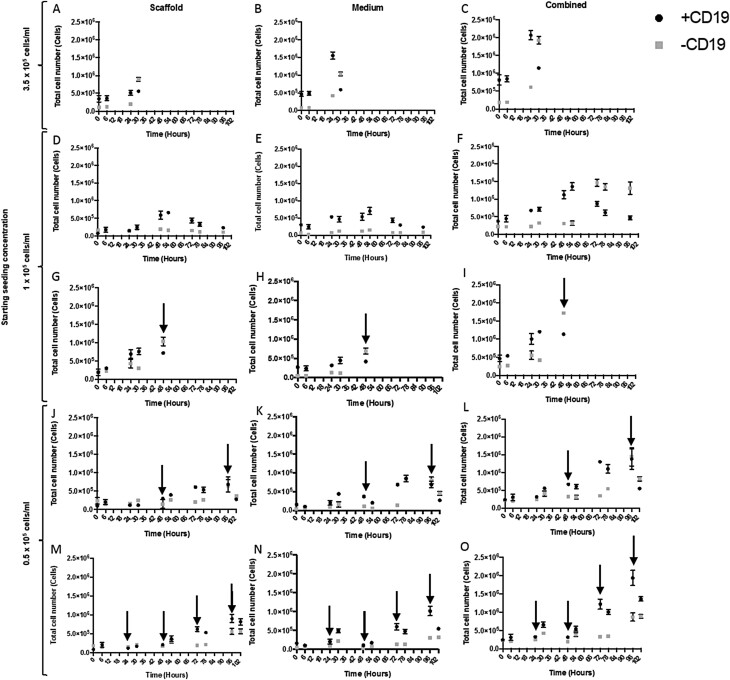
Initial seeding density and long-term culture of TK6 (CD19^+^) cells, co-cultured with pre-cultured AlgiMatrix™ scaffold. HS-5 (CD19^−^) cells seeded onto AlgiMatrix™ scaffolds at a density of 2.5 × 10^5^, incubated for 24 h, complete medium change conducted, incubated for a further 144 h with a 50% medium change every 48 h. TK6 cells were then combined at an initial concentration of 3.5 (A, B, C), 1 (D, E, F, G, H, I), or 0.5 × 10^5^ cells/ml (J, K, L, M, N, O). A 0% (A, B, C, D, E, F) or 50% (G, H, I, J, K, L, M, N, O) medium change was conducted every 0 h (A, B, C, D, E, F), 24 h (M, N, O), or 48 h (G, H, I, J, K, L) over a 102-h period indicated by an arrow. Twice over 24 h, 6 h apart, cells were collected from the medium (B, E, H, K, N) and dissolved scaffold (A, D, G, J, M), counted, and stained separately with CD19 antibody. Percentage positive of cells was assessed by flow cytometry for total CD19^+^ and CD19^−^ in the scaffold and medium giving a combined total for cells in the well (C, F, I, K, O) (50 000 events, *n* = 3).

To investigate the required initial seeding density and exponential growth phase of TK6 cells for drug/chemical dosing of actively proliferating cells within a scaffold co-culture, HS-5 cells were seeded onto AlgiMatrix™ and incubated using the protocol described previously. TK6 cells were then directly added to each scaffold at an initial seeding density of 0.5, 1, and 3.5 × 10^5^ cells/ml. A medium change was either not conducted or conducted every 24 or 48 h over a 102-h period following the addition of the TK6 cells. TK6 were distinguished from HS-5 in co-culture via flow cytometry analysis of CD19 positivity; thus, data are presented in Fig. 7 as CD19^+^ (TK6) and CD19^−^ (HS-5).

Scaffolds seeded with an initial TK6 cell density of 3.5 × 10^5^ cells/ml, saw combined CD19^+^ from the scaffold (Fig. 7A) and medium (Fig. 7B) increase over an initial 24-h period (Fig. 7C) to a combined density of 2.0 × 10^6^ cells. However, after a further 6 h, the combined CD19^+^ cell population decreased, whilst CD19^−^ cells surpassed CD19^+^ and reached ~2.0 × 10^6^ by 36 h. Due to this shift in CD19^−^ predominance over CD19^+^ cells, the experiment was terminated, and a medium change was not conducted. This result indicated that a lower seeding density of TK6 was required for culture past 24 h.

A seeding density of 1 × 10^5^ cell/ml was then performed (Fig. 7D–F), which demonstrated CD19^+^ cells entering an exponential growth phase at 24 h, with a steady increase up to 1.4 × 10^6^ total combined cell number at 54 h. Over the same time period, CD19^−^ cells remained <2.5 × 10^5^. However, after a further 24 h of culture, the combined CD19^−^ cell population increased and surpassed that of a decreasing CD19^+^ population. The shift to CD19^−^ predominance was seen for the remainder of the experiment, with CD19^+^ cells steadily decreasing from 1.4 × 10^6^ to 5 × 10^5^ cells between 54 and 96 h with CD19^−^ cells remaining at ~1.3 × 10^6^. As neither of these conditions had incorporated a medium change, and it was thought this might affect CD19^+^ (TK6) growth, the introduction of a medium change was then investigated.

An initial seeding density of 1 × 10^5^ CD19^+^ (TK6) cells/ml was performed followed by a 50% medium change at 24 h (Fig. 7G–I). This medium change extended the duration that CD19^+^ cells proliferated (0–30 h compared with 3.5 × 10^5^ cells/ml at 0–24 h), with CD19^−^ cells remaining <2.5 × 10^5^. However, a medium change at this time point (24 h) caused a decrease in CD19^+^ and an increase in CD19^−^ cells, at ~30–48 h. These data indicated that a lower seeding density would be necessary but with added medium changes.

An initial seeding density of 0.5 × 10^5^ cell/ml was investigated with a medium change every 24 h (Fig. 7M–O) and 48 h (Fig. 7J–L). With medium changes every 24 h, CD19^+^ cells remained below 7 × 10^5^ combined cells until hour 54, at which point they entered an exponential growth phase up to 96 h (5 × 10^5^ to 2 × 10^6^ total cells) with CD19^−^ cells remaining at <5 × 10^5^ up to 96 h. At 102 h, CD19^+^ cell numbers decreased and CD19^−^ cells increased in cell number but did not predominate. The introduction of a medium change at every 48 h was also attempted (Fig. 7J–L), however, CD19^+^ cells decreased at 96 h and were predominated by CD19^−^ cells. However, the start of the exponential growth phase occurred at the same time period (54–96 h) for medium changes at both 24- and 48-h intervals. The proliferation of CD19^+^ (TK6) cells in the medium and scaffold was at the same rate irrespective of the compartment they resided in, however, an increase in TK6 within the scaffold was required before an exponential increase in the medium was seen. These data indicated that TK6 seeded at an initial density of 0.5 × 10^5^ cell/ml with a 50% medium change every 24 h produced an exponential growth phase, suitable for compound dosing, between 54 and 96 h (Fig. 7O).

**Figure 8.**
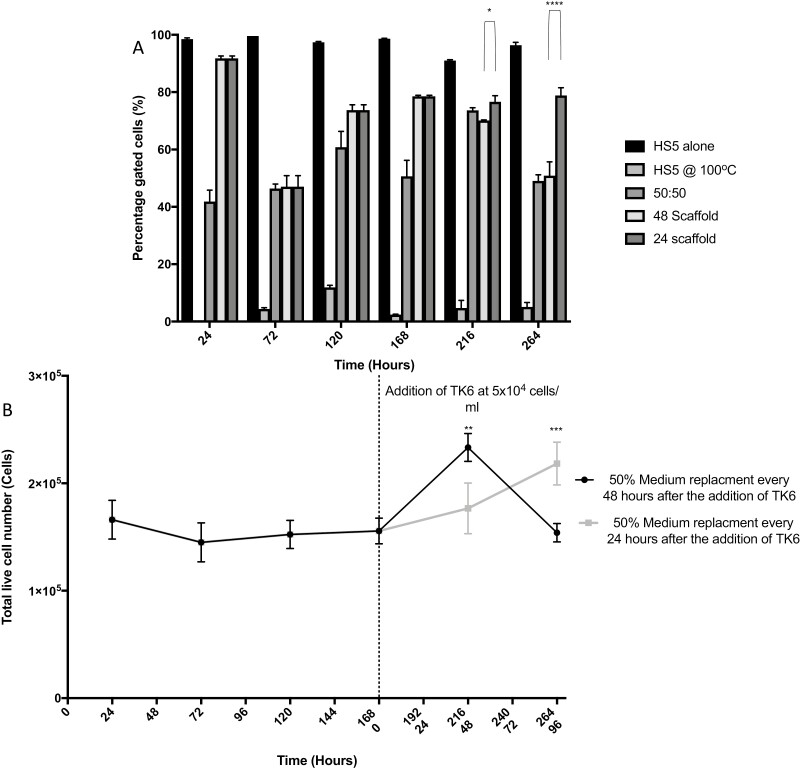
Viability of HS-5 cells seeded onto an AlgiMatrix™ scaffold with the addition of TK6. HS-5 cells seeded onto AlgiMatrix™ scaffolds at a density of 2.5 × 10^5^, incubated for 24 h, complete medium change conducted, incubated for a further 144 h with a 50% medium change conducted every 48 h. At each 48-h time point, scaffolds were harvested and assessed for total cell number (TB) and viability. After 168 h of incubation, TK6 cells were added at a concentration of 5 × 10^4^ cells/ml into a 0.25 µm well insert residing within each scaffold well. A 50% medium change is conducted every 24 or 48 h. Each time point was compared to a dead (HS-5 incubated at 100°C for 10 min), live (HS-5 cells at a viability of >90%), and 50:50 (dead:live) control for percentage positivity (A). This percentage positivity was then used to calculate the total number of live HS-5 cells in each condition (B) (50 000 events, *n* = 3). Significant differences between samples were calculated using a two-way ANOVA followed by a Dunnett’s test. The *P* values are indicated by **P < .01, ***P < .001, ****P < .0001.

The establishment of an initial seeding density and protocol for the longevity of both cell lines allowed the investigation of HS-5 viability and cell number (Fig. 8) under the influence of TK6 co-culture and medium changes. After an initial incubation (168 h) of HS-5 onto the AlgiMatrix scaffold, HS-5 cell viability (alone) remained high (Fig. 8A; ~100%) as assessed by flow cytometry. TK6 cells (5 × 10^4^ cells/ml) were added in a well insert and co-cultured with the HS-5-seeded scaffolds; a 50% medium change was conducted either every 24 or 48 h after TK6 addition. The 24-h group generally maintained 80% HS-5 cell viability over the entire co-culture, whereas the 48-h group dropped to around 50% towards the end of the experiment, inferring that TK6 had minimal influence,  but the frequency of medium change did affect cell counts over a longer term.

Total live HS-5 cell number was calculated using the viability from flow cytometry with total cell number via TB (Fig. 8B). Live HS-5 cell number was maintained between 0 and 168 h, however, TK6 addition encouraged an increase in live HS-5 cell numbers, with 24-h medium changes promoting an HS-5 cell number increase, whereas 48-h medium changes did not. These outcomes reinforce earlier results in identifying the need for a medium change every 24 h.

### Dose-escalation of known genotoxic, *in vivo* positive, and non-genotoxic compounds within the 2D *in vitro* MN assay

**Figure 9.**
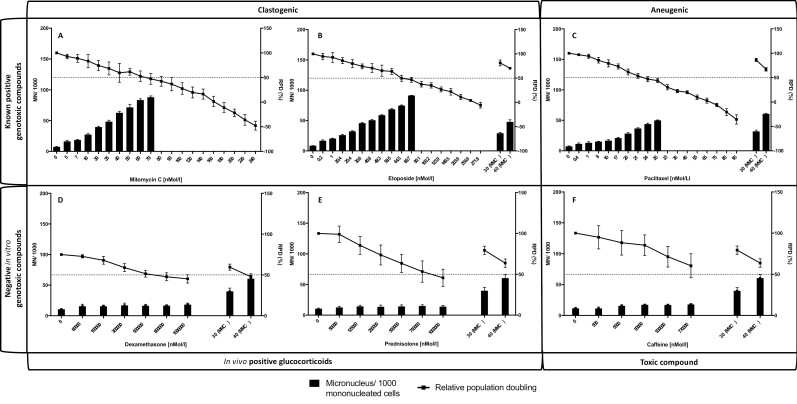
The RPD and MN induction in TK6 cells after treatment with known positive and negative genotoxic compounds in the 2D assay. TK6 cells were treated for a 48-h treatment period (24-h direct + 24-h recovery) with the concentration of MMC (A), etoposide (B), paclitaxel (C), dexamethasone (D), prednisolone (E), or caffeine (F) to create a dose range inducing an RPD between 100% and 0%. RPD was calculated utilizing OECD guidelines with the dotted line representing an RPD of 50%. MN were assessed in 2000 mononucleated cells per concentration, per repeat, and expressed as micronuclei per 1000 mononucleated cells. A known positive internal control (MMC) was run with each compound to ensure that the assay had been successful.

In accordance with OECD guideline 487, a total of six compounds were analysed for RPD and the induction of MN using the 2D *in vitro* MN assay, to assess the appropriate concentration range for use within the 3D model. A range of concentrations including those below the accepted RPD limit of 50% were seen as necessary to account for a lack of bioavailability of the compound to cells within a scaffold, through detoxification and/or interaction with the ECM [16]. The six compounds consisted of two clastogenic agents (MMC and etoposide) and one aneugenic compound (paclitaxel) as positive controls, two glucocorticoids known to be weakly positive *in vivo* (dexamethasone and prednisolone), and one known toxic but non-carcinogenic compound (caffeine; negative control; Fig. 9). A summary of the concentrations which were taken forward for testing within the 3D model can be seen in Table 1.

**Table 1.** Summary of the concentrations of known positive, negative, and *in vivo* positive compounds which gave an RPD range between 100% and 0% in TK6.

Group	Compound	Doses identified within the regulatory 2D MN assay for addition to the 3D model	
		Concentration (nmol/L)	RPD (%)
Clastogen	Mitomycin C	0	100
		7	89
		35	68
		60	53
		90	37
		140	16
	Etoposide	0	100
		1	93
		40.8	71
		64.5	50
		100.2	35
		203.9	11
Aneugen	Paclitaxel	0	100
		1	94
		17	73
		21	54
		33	30
		45	20
Glucocorticoid Weak *in vivo* positive	Dexamethasone	0	100
		1 × 10^5^	91
		3 × 10^5^	79
		5 × 10^5^	67
		8 × 10^5^	64
		1 × 10^6^	60
	Prednisolone	0	100
		5 × 10^4^	99
		2.5 × 10^5^	74
		5 × 10^5^	63
		7.5 × 10^5^	53
		1 × 10^6^	46
Toxic non-carcinogens	Caffeine	0	100
		5 × 10^2^	95
		5 × 10^3^	88
		5 × 10^4^	85
		5 × 10^5^	71
		7.7 × 10^5^	60

TK6 cells were treated for a 48-h treatment period (24-h direct + 24-h recovery) with RPD calculated utilizing OECD guidelines. MN were assessed in 2000 mononucleated cells per concentration, per repeat, and expressed as micronuclei per 1000 mononucleated cells.

All three genotoxic compounds, as expected, demonstrated a dose-dependent increase in MN induction alongside a dose-dependent decrease in RPD in the 2D assay (Fig. 9A–C). MN were scored only for doses achieving an RPD of 55% ± 5% or above, and significant increases in MN were achieved at the lowest doses for all three compounds (MMC, *P* = .0018; etoposide, *P* = <.0001; paclitaxel, *P* = .0213). In the 2D assay, MN were not scored above 70 nmol/L for MMC (47% RPD), 69.7 nmol/L for etoposide (46% RPD), and 24 nmol/L for paclitaxel (47% RPD). Doses that calculated a ‘minus’ RPD value were not taken forward into 3D co-culture models. Doses of 30 and 40 nmol/L for MMC were identified for use as a positive control in subsequent MN assays.

Based on the data produced in the 2D MN assay (Fig. 9A–C), the following doses produced an RPD range spanning 100%–80%, 80%–60%, 60%–40%, 40%–20%, 20%–0%, respectively, and were used for 3D scaffold co-culture MN analyses: MMC—7, 35, 60, 90, and 140 nmol/L; etoposide—1, 40.8, 64.5, 100.2, and 203.9 nmol/L; paclitaxel—1, 17, 21, 33, and 45 nmol/L.

Dexamethasone, prednisolone, and caffeine were similarly assessed in the 2D MN assay (Fig. 9D–F). As expected, all three compounds did not achieve a genotoxic dose, up to and including a maximum soluble dose of 7.7 × 10^5^ nmol/L (caffeine) and 1 × 10^6^ nmol/L (dexamethasone and prednisolone). However, unlike dexamethasone and caffeine, prednisolone achieved an RPD of 55% ± 5% at the highest concentration of 1 × 10^6^ nmol/L (46%). The MMC-positive control, however, did confirm a significant increase in MN when compared to the vehicle control, validating the assay and confirming that dexamethasone, prednisolone, and caffeine can be deemed as non-genotoxic within the 2D MN assay. As an RPD range spanning 100%–80%, 80%–60%, 60%–40%, 40%–20%, 20%–0% was not identified, five concentrations (Table 1), up to and including the maximum concentration were taken forward for testing within the 3D MN co-culture model.

### Genotoxic assessment of known genotoxic, *in vivo* positive, and non-genotoxic compounds within the 3D *in vitro* bone marrow model

Each concentration of compound identified for use within the 3D model (Table 1) was applied to the cells using the protocol set out in Fig. 1. Once the 48-h treatment period had concluded (24-h direct treatment + 24-h recovery) [14], MN induction was assessed in cells of the medium and scaffold for a combined total before comparison to the results found within the 2D assay (Table 2).

**Table 2.** Comparison of the MN induced within the 2D assay and 3D model of known positive, negative, and weak *in vivo* positive compounds.

Group	Compound	Concentration (nmol/L)	Micronucleus/1000 mononucleated cells within the 2D regulatory assay and 3D model	
			2D *in vitro*	Combined 3D scaffold and medium
Clastogen	Mitomycin C	0	7	15
		7	18	25
		35	48	29
		60	83	59
		90		77
		140		95
	Etoposide	0	8	15
		1	20	24
		40.8	51	32
		64.5	75	46
		100.2		51
		203.9		54
Aneugen	Paclitaxel	0	7	15
		1	13	34
		17	21	38
		21	37	41
		33		46
		45		52
Glucocorticoid Weak *in vivo* positive	Dexamethasone	0	10	15
		1 × 10^5^	15	23
		3 × 10^5^	17	27
		5 × 10^5^	16	27
		8 × 10^5^	16	32
		1 × 10^6^	18	32
	Prednisolone	0	10	15
		5 × 10^4^	12	24
		2.5 × 10^5^	13	29
		5 × 10^5^	14	31
		7.5 × 10^5^	15	31
		1 × 10^6^	14	32
Toxic non-carcinogens	Caffeine	0	11	16
		5 × 10^2^	11	20
		5 × 10^3^	15	20
		5 × 10^4^	17	18
		5 × 10^5^	17	19
		7.7 × 10^5^	18	21

Within both assays, a 48-h treatment period (24-h direct + 24-h recovery) was conducted. MN were assessed in 2000 mononucleated cells per concentration, per repeat, and expressed as micronuclei per 1000 mononucleated cells. Within the 2D assay, MN were assessed in TK6 of the medium, whereas MN in the 3D model is a combined total of those found in the medium and harvested scaffold. Concentrations without a MN score within the 2D assay, in line with OECD guidelines, could not be assessed for micronuclei as an RPD <55% ± 5% was achieved.

**Figure 10.**
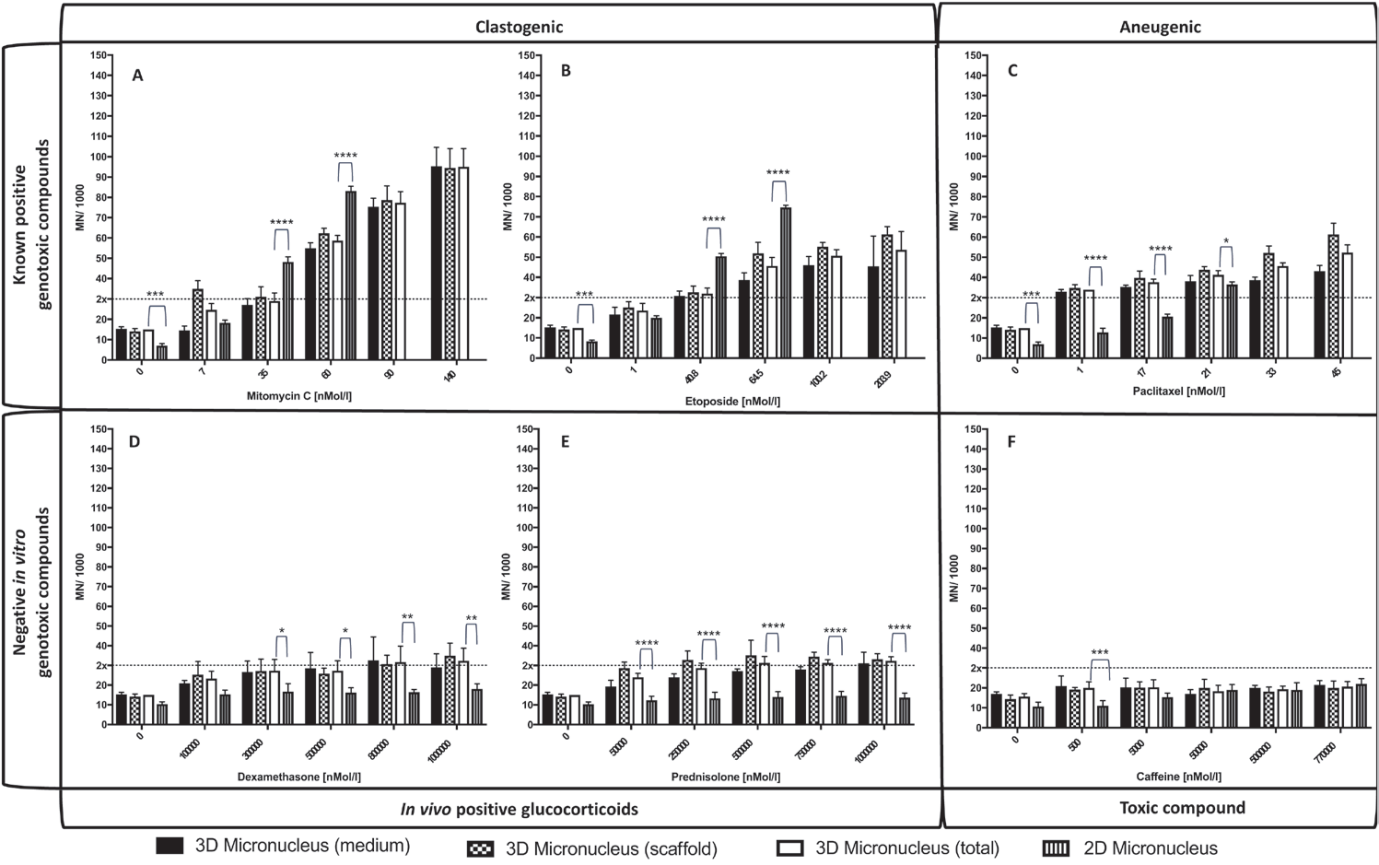
The MN induction from cells within the novel 3D BM model was treated with known positive and negative genotoxic compounds. TK6 and HS-5 cells within the 3D model were treated for a 48-h treatment period (24-h direct + 24-h recovery) with the concentration of MMC (A), etoposide (B), paclitaxel (C), dexamethasone (D), prednisolone (E), or caffeine (F). Micronuclei were assessed in 2000 mononucleated cells within both the medium and scaffold per concentration, per repeat before being combined to give a total MN count for each well, and expressed as micronuclei per 1000 mononucleated cells. The dotted line represents a total MN count 2× the vehicle control within the 3D model. Concentrations without a 2D MN count induced an RPD <55% ± 5% so were not scored for micronuclei. Significant differences in MN between the 2D and 3D counts were indicated with *P < .05, **P < .01, ***P < .001, ****P < .0001.

Cells in 3D cultures were assessed for viability and cytotoxicity using CD19 positivity within flow cytometry as a marker of live TK6 cells as described above. Interestingly, TK6 cell proliferation in treated cultures remained in exponential phase such that calculated RPD values were in excess of 100% throughout. Furthermore, in line with *in vivo* observations of BM protection of haematopoietic and stem cells from chemotherapy, TK6 homed to the interior of the scaffold. HS-5 cells remained low as observed in Fig. 7M–O, thus all cells were scored for MN in 3D cultures. All three genotoxic agents induced statistically significant increases in MN in 3D assays. MMC and etoposide generally induced MN at levels equivalent to or lower than those produced in the 2D assay, whereas MN from paclitaxel were higher or equivalent to the 2D assay. Additionally, there was a dose-dependent increase of MN within the 3D model at doses that were not possible to be scored in the 2D model (Fig. 10A–C). MMC induced a significant increase in total MN within the 3D model at 60 nmol/L (59 MN/1000 mononucleated cells; *P* < .05) compared with the 2D assay at 7 nmol/L (25 MN/1000 mononucleated cells; *P* = .0018; Fig. 10A). There was a significant increase in MN within the 2D assay compared to the 3D model at concentrations of 35 nmol/L (48 vs. 29 MN/1000 mononucleated cells; *P* < .0001) and 60 nmol/L (83 vs. 59 MN/1000 mononucleated cells; *P* < .0001). These data suggest that the 3D model can tolerate a higher concentration of, or is less sensitive to, MMC than the 2D assay.

Etoposide induced a significant increase in MN at 40.8 nmol/L (32 MN/1000 mononucleated cells; *P* < .01) in the 3D assay compared with the 2D assay at 0.2 nmol/L (20 MN/1000 mononucleated cells; *P* < .0001; Fig. 10B). In a similar trend to MMC, concentrations of 40.8 and 64.5 nmol/L induced significantly more MN within the 2D assay (51 and 75 MN/1000 mononucleated cells; *P* < .0001) compared with the 3D model (32 and 46 MN/1000 mononucleated cells), inferring that a higher concentration of both MMC and etoposide is required within the 3D model to replicate the same level of genotoxicity seen within the 2D assay.

Paclitaxel produced a significant increase in MN cells at a concentration of 1 nmol/L (34 MN/1000 mononucleated cells; *P* < .0001), however, within the 2D assay, statistically significant increases in MN cells required 4 nmol/L (*P* = .0213; Fig. 10C), suggesting that paclitaxel within the 3D model was more genotoxic. Furthermore, this trend continued in a dose-dependent manner as MN induction in the 2D assay decreased compared to its 3D counterpart (Fig. 10C).

As expected, neither the 2D assay nor the 3D model demonstrated any induction of MN in the caffeine negative control (Fig. 10F). Neither assay was capable of inducing a statistically significant increase of MN at twice that of the vehicle control (16 MN/1000 mononucleated cells), with MN remaining around 20 MN/1000 mononucleated cells. This result infers that caffeine is non-genotoxic in both the 2D assay and the 3D model.

However, for the glucocorticoids dexamethasone and prednisolone, the 3D model was able to demonstrate genotoxic events that the 2D assay failed to achieve suggesting that the 3D model may offer a predictability of rodent outcomes for such compounds (Fig. 10D and E). Dexamethasone induced statistically significant increases of MN above the vehicle control at 8 × 10^5^ nmol/L (*P* < .05) and statistically significant increases over the 2D assay for concentrations of 3 × 10^5^, 5 × 10^5^ (*P* < .05), and 8 × 10^5^ and 1 × 10^6^ nmol/L (*P* < .01). Unlike the 2D assay which did not identify a genotoxic dose, the 3D model produced 32 MN/1000 mononucleated cells at both dexamethasone concentrations of 8 × 10^5^ and 1 × 10^6^ nmol/L, which was twice that of the vehicle control (0 nmol/L had 15 MN/1000 mononucleated cells; Fig. 10D). These data suggest that within the 3D model, dexamethasone is weakly genotoxic at high concentrations.

Prednisolone was capable of inducing a statistically significant increase in MN over the vehicle control from the lowest dose of 5 × 10^4^ nmol/L (*P* < .01; Fig. 10E). Similar to dexamethasone, whilst vehicle controls were at similar levels, prednisolone significantly increased MN within the 3D model compared to the 2D assay for all doses investigated (*P* < .0001). Whilst the 2D assay indicated prednisolone was non-genotoxic, within the 3D model, concentrations of 5 × 10^5^, 7.5 × 10^5^, and 1 × 10^6^ nmol/L induced 31, 31, and 32 MN/1000 mononucleated cells, respectively, which were twice the vehicle control of 15 MN/1000 mononucleated cells. This result suggests that prednisolone is also marginally genotoxic at a concentration above 5 × 10^5^ nmol/L within the 3D model.

## Discussion

The *in vitro* 2D regulatory MN assay is commonly used in the pharmaceutical industry to evaluate the genotoxic potential of a compound before conducting *in vivo* tests [7]. However, compounds such as the glucocorticoids (dexamethasone and prednisolone), which have not been seen as carcinogenic in the clinic and were negative for genotoxicity within the 2D regulatory *in vitro* MN assay, were marginally positive within the (3D) *in vivo* rodent BM MN assay [8–10]. Therefore, an optimized co-culture of cell lines seeded upon an AlgiMatrix™ scaffold was used to assess the genotoxicity of known positive, negative, and *in vivo* positive only compounds compared to historical *in vivo* rodent data, with the aim to bridge the gap between current assays and better predict *in vivo* outcomes. The eventual outcome of the model was to create an environment that simulated the *in vivo* human, reducing the need for animal testing and with the aim to create a more ‘human-relevant’ test bed; therefore, human cell lines were used within the model. In this study, we provide initial evidence that the use of a simple *in vitro* model of the BM can more accurately predict the genotoxic outcome of a compound within a rodent *in vivo* study.

### Establishing a new 3D model for micronucleus assessment

The AlgiMatrix™ system consists of a freeze-dried alginate scaffold, which is rehydrated with concentrations of firming buffer with/without cells depending on the desired internal structure [17]. The human BM consists of pores around 100–250 µm, which was achieved with 50% firming buffer within the AlgiMatrix; pore size has been found to have a considerable impact on cellular differentiation and proliferation of the cellular compartments within the BM [18]. A pore of 100–200 µm was also found to be beneficial for cellular morphology, proliferation, and osteogenic differentiation of human mesenchymal stem cell (MSCs) [19], signifying that the pore size produced within a 50% solidified scaffold of 100 and 150 µm would be *in vivo* relevant and appropriately support the HS-5 human stromal cell line.

The constituents of the firming buffer supplied with the AlgiMatrix™ system are unknown and appeared to have an effect on the viability of the HS-5 cells seeded within. However, this viability decrease can be abrogated by reducing the firming solution in the medium by 90% on Day 2, combined with a medium change 48 h to accommodate the expanding cell number. This finding correlates with the use of a divalent cation, such as CaCl_2_, used as a crosslinking agent in alginate gels [20], where even at low levels, a decrease in viability can be seen requiring further dilution and incubation for increased viability [21–23]. Given these observations, a 50% AlgiMatrix™ scaffold simulated the *in vivo* ECM more closely and therefore was utilized for further study.

HS-5 cells were seeded onto the AlgiMatrix™ scaffold at a total density of 2.5, 5, 7.5, and 10 × 10^5^ cells/scaffold before being transferred to a 12-well plate. An initial density of 2.5 × 10^5^ cells/scaffold appeared to maintain the same density throughout the experiment without loss. Previous work within our laboratory suggested that Ki67 expression does not align well with HS-5 proliferation in 2D culture (S. Andrews *et al.*, in preparation), however, in 3D models Ki67 does correlate with viability and proliferation over a 168-h period, despite a high proportion of cells remaining in the G0 stage of cell cycle throughout. Conversely, each cell density, after a 168-h period, increased in cell number, plateaued/decreased viability, and decreased Ki67 expression. As previous literature has highlighted, regarding the stall of BM cells within G0, if BM stromal cells do not have contact with HSCs for an extended period, they will reduce in viability and cell number to conserve cellular numbers in dwindling supplementation [24–26]. In consideration of this, the original, lower seeding density of 2.5 × 10^5^ total cells seeded onto an AlgiMatrix™, correlates with the current *in vivo* BM literature, with a maximum seeding time of 168 h before the introduction of an additional haematopoietic cell line.

The human B lymphoblastoid cell line, TK6, is used routinely in 2D genotoxicity studies within the pharmaceutical industry. This cell line is used due to its proliferation index (12- to 15-h doubling time), p53 competency, human origin, and ease of culture allowing a rapid 7-day analysis of potential therapeutic compounds [27]. Due to these advantages, TK6 was considered the best candidate cell line for addition to the AlgiMatrix™-cultured HS-5 to compare MN induction in 2D with our 3D model and subsequently with historic *in vivo* data. As mentioned previously, BM stromal stem cells remain in a quiescent state until the addition of HSCs which then increase the MSC’s viability, proliferation, and cell number. The model’s ability to simulate this *in vivo* behaviour, was once again reinforced with the addition of TK6, in a 0.2 µm pore culture insert, to HS-5 seeded onto AlgiMatrix™ scaffolds for 7 days (Fig. 8). This result infers that the addition of soluble factors, not just cell–cell contact, is sufficient to induce an increase in HS-5 in alignment with the *in vivo* literature.

The *in vitro* and *in vivo* MN assay requires the active proliferation of the HSC compartment for the formation of MN. Utilizing CD19 as a positive indicator of TK6 only (Fig. 6; Krüger *et al.* [28]), TK6 cells were added to AlgiMatrix™-cultured HS-5, and an exponential phase of CD19^+^ cells was observed, which closely corresponds with the timeframe seen in the *in vitro* MN assay, allowing for the comparison between 2D and 3D MN induction *in vitro*. The eventual reduction of the CD19 marker indicates membrane degradation of dead or dying cells through reduction in medium supplementation not necessarily an increase in CD19^−^ HS-5 cells [29].

### Initial dose-escalation for identification of concentrations for use within a 3D model of the bone marrow

Utilizing the 2D *in vitro* MN assay set out by the OECD [13], a dose-escalation was conducted of known positive (MMC, etoposide, and paclitaxel), negative (caffeine), and weak positive *in vivo* (dexamethasone and prednisolone) compounds to firstly identify an RPD of 55% ± 5%, up to a maximum concentration of 1 mM or maximum soluble concentration, with MN counted in doses which gave an RPD >50%.

Concentrations of MMC, etoposide, and paclitaxel which achieved an RPD of >55% ± 5% and relative MN count at least twice that of the vehicle control was compared to, and agreed with, historical literature confirming these positive genotoxic compounds [30–33]. In agreement with historical literature, at a maximum soluble concentration or 1 mM, dexamethasone, prednisolone, and caffeine did not exceed the RPD limit of 55% ± 5% or MN induction twice that of the vehicle control, confirming these as negative genotoxicants [12, 34, 35].

It has been shown that, within alternative 3D multicellular models, a higher concentration of the compound is usually required than the 2D monoculture counterpart, due to a reduction in bioavailability (adherence to scaffold), detoxification, and dynamic dosing [15, 36]. Therefore, the present study is the first to identify an RPD dose range spanning 100%–0% for MMC, etoposide, and paclitaxel. It was intriguing to note that none of these doses caused cytotoxicity in the 3D model, leading to calculated RPD of >100% throughout, suggesting agreement with the concept of reduced bioavailability.

### Genotoxicity of compounds within a simple 3D multicellular model of the bone marrow

Utilizing the same treatment timeline as the 2D *in vitro* MN assay, concentrations identified which induced an RPD range of 100%–80%, 80%–60%, 60%–40%, 40%–20%, 20%–0% were added to the 3D model. To maintain cellular conditions within the scaffold and wash off compound after the initial 24-h direct treatment, only the medium around the scaffold was harvested, the collected cells were washed and reseeded; therefore, some compounds may have still been present within the scaffold during the recovery period. However, when each compound was investigated for its relevant half-life within *in vitro* culture, a half-life of <24 h was identified [MMC (50 min), etoposide (1.5 h), paclitaxel (13 h), dexamethasone (190 min), prednisolone (60 min), and caffeine (5 h)] [37–43], inferring that even if the compound was still present within the scaffold it would have likely been in trace amounts and have limited further effect on the cells within.

The total MN induction of the clastogenic compound MMC, within the 3D model, was found to be less genotoxic than at the same concentration seen within the 2D assay. Studies by Niikawa *et al.* [44] and Bowen *et al.* [45] in Swiss albino mice and rats found that when 1 mg/kg of MMC was administered, the resulting unbound plasma concentration was at 45 ng/ml, with MN induction at this concentration 8-fold higher than the vehicle control. However, at the highest concentration tested within the 3D model of 46.8 ng/ml (140 nmol/L), a 6-fold increase in MN was seen, suggesting that in a similar manner to the rodent, the administered compound had been reduced either through detoxification or binding to the scaffold, but the outcome closely mimicked the high concentration of compound required for genotoxicity within the *in vivo* environment [15, 36]. However, at a maximum scorable dose within the 2D *in vitro* assay of 20 ng/ml, a 7-fold increase in MN from the vehicle control was seen, highlighting the overestimation of genotoxicity within a 2D monoculture.

In a similar manner to MMC, etoposide induced a greater level of MN within the 2D *in vitro* assay than the 3D model. In agreement with the findings, work by Turner *et al.* [46] found that when mice were administered 1 mg/kg of etoposide, the resulting unbound plasma concentration was 10 000 ng/ml; at this concentration within the plasma, a 25-fold increase in MN was seen. The highest concentration trialled within the 3D model was 120 ng/ml (209.9 nmol/L) which induced a 3-fold increase in MNs. At the highest scorable dose within the 2D assay of 38 ng/ml (64.5 nmol/L), which is 263 times less concentrated than that seen in the *in vivo* plasma, a MN induction 11-fold higher than the vehicle control was induced.

The MN induction seen with paclitaxel, in contrast to MMC and etoposide, appeared to be greater in the 3D model when compared to the same concentration within the 2D *in vitro* MN assay. However, the background level of MN within the 3D assay was 2-fold higher than the 2D assay, so when this was corrected for, the data inferred that 3D once again induced less MN than 2D. Studies by Zaid *et al.* [47] and Rabah *et al.* [48] found that a dose of 0.6 mg/kg paclitaxel gave an unbound blood plasma concentration of 53 ng/ml, resulting in an MN induction 16-fold higher than the vehicle control. The highest concentration utilized within the model was 38.4 ng/ml (45 nmol/L) which induced a 3-fold increase in MN.

The non-genotoxic compound caffeine did not induce a genotoxic level of MN within either the 3D model or 2D assay, which agreed with historical studies conducted within rodents confirming its non-genotoxic status [49, 50]. The results seen within the 3D *in vitro* model with concentrations of known positive and negative genotoxicants seemed to mimic the increase in concentration needed for MN induction within the *in vivo* rodent, whilst highlighting the overestimation which occurs in the 2D assay. However, as the free concentration of each compound was not assessed within the model in this preliminary study, it cannot be confirmed that the same level of detoxification and bio-reduction have seen within the *in vivo* rodent was present in the model. The literature infers that the BM is in fact metabolically competent [51], and data within our laboratory suggest the metabolic capacity of HS-5 cells (Vernon *et al.*, unpublished data), so this requires further analysis. Nevertheless, the model was able to better predict the outcome of each compound within *in vivo* rodent assays compared to the 2D assay, so the model was tested for its ability to accurately predict dexamethasone and prednisolone as weak *in vivo* positive compounds.

When dexamethasone was assessed for MN induction within the 2D *in vitro* MN assay, a genotoxic dose twice that of the vehicle control was not achieved up to and including a maximum dose of 1 mM. However, within the 3D model, a concentration of 8 × 10^5^ nmol/L (3.1 × 10^5^ ng/ml) and higher induced a 2-fold induction of MN. In agreement with this finding, work by Li *et al.* [52] and Singh *et al.* [53] found that in Swiss albino mice given 1 mg/kg dexamethasone, an unbound plasma concentration of 287.5 ng/ml was found which induced a 3-fold increase in MN compared to the vehicle control, which was not seen in the 2D assay. Dexamethasone at 8 × 10^5^ nmol/L (3.1 × 10^5^ ng/ml) is 107 times more concentrated than the plasma levels seen within the *in vivo* rodent. However, as previously highlighted, if compounds are bio-reduced within the 3D model, the actual concentration available to the cells could be at a similar level as seen *in vivo* and therefore requires further validation for inducing a similar level of MN to the *in vivo* rodent. When prednisolone was assessed in the 2D assay, similar to dexamethasone, it did not induce MN twice that of the vehicle control at any concentration up to and including 1 mM. However, within the 3D model, a concentration of 5 × 10^5^ nmol/L (1.8 × 10^5^ ng/ml) or higher was found to induce a 2-fold increase in MN. This result agrees with studies put forward by Meno-Tetang *et al.* [54] and Hayes *et al.* [8], which found that concentrations of unbound prednisolone, between 8 × 10^4^ and 1.8 × 10^5^ ng/ml, gave a consistent MN induction twice that of the vehicle control which they did not observe within their 2D *in vitro* assay. Our results suggest that similar doses of prednisolone in the 3D model induced MN to levels observed in rodents *in vivo*, which were not observed within our 2D MN assay.

### Future development of the model

Whilst a battery of tests is required to definitively assign genotoxicity to unknown compounds, the observation that false-positives in the rodent BM MN assay are rare [9] has highlighted the importance for further investigation and understanding of the underpinning mechanisms leading to *in vivo* positive compounds [55]. An assay that can accurately and consistently predict this outcome, and control for variables currently known to influence MN induction, has the capacity to bridge the gap between *in vitro* and *in vivo* testing. Expanding this assay through testing other candidate *in vivo* positive compounds, such as glucocorticoid receptor agonists, as well as carcinogens such as urethane, procarbazine, and benzene, the anti-inflammatory sulfapyridine and the opioid morphine may offer capacity to better understand the mode of action if shown to be more widely predictive [56]. Tweats *et al.* noted that many such compounds interfered with cell cycle kinetics [56], so novel compounds with this capacity may require more extensive testing, with the inclusion of 3D models such as these.

Our 3D model offers a cost-effective and simple to construct genotoxicity assay utilizing haematopoietic and stromal cell lines from human BM which accurately predicted *in vivo* positive outcomes for glucocorticoids where 2D *in vitro* assays failed. We have previously demonstrated genotoxicity parallels between *in vitro* chemotherapy treatment of HS-5 cells with chemotherapy-treated patient samples, further validating the use for ‘humanised’ models for modelling patient and/or *in vivo* human exposure outcomes [57]. In developing this 3D model from human cell lines, the model reduces the scope for genetic variability which may occur from primary cells, has consistent human-relevant metabolism (which may counteract the proposed inadequacies of rat S9 *in vitro*), can be controlled for fluctuations in temperature and may offer an accessible format for others to adopt. These benefits may help confirm and address the current understanding of the mode of action of *in vivo* positive compounds.

In conclusion, this is the first 3D model utilizing human cell lines to correctly predict the genotoxicity of known positive, negative, and weakly positive *in vivo* compounds in an *in vitro* 3D model when compared to historical rodent data. This preliminary study, using a simple co-culture model of cell lines upon an artificial ECM, will now enable the exploration of the mechanism(s) behind such compounds, allowing a more accurate drug discovery pathway with a possible reduction in unnecessary animal testing [55].
